# An Account of a New Method of Treating Diseases of the Joints of the Knee and Elbow, in a Letter to Mr. Percival Pott

**Published:** 1783

**Authors:** 


IX. *An account of a new method of treating diseases of the joints of the knee and elbow, in a letter to Mr.* Percival Pott, *by* H. Park, *of Liverpool, one of the surgeons of the hospital.* 8vo. Johnson, *London*, 1783. 51 pages. 1 s. 6d.

SCROPHULOUS affections of the joints; collections of pus in the articular cavities; gun-shot wounds and compound fractures of the joints; nay, even the most simple, penetrating wounds, however favourable may be the termination in some few cases, are nevertheless, in spite of all the modes of obtaining relief hitherto discovered, but too frequently productive of such a train of evils, as to terminate at length in the destruction of the unhappy sufferer, unless prevented by the timely removal of the limb. To alleviate in some degree these evils, and to avert some of the dreadful consequences, is the design of the work before us. The ingenious author clearly shews, that in some of the affections of the knee and elbow, in which amputation has hitherto been deemed indispensably necessary. Sur-

Surgery has yet another reſource, by which the limbs of perſons under the above circumſtances may be preſerved, with ſuch a ſhare of the motions which nature had originally allotted to them, as to be conſiderably more uſeful than any invention which art has hitherto been able to ſubſtitute in their ſtead. This reſource conſiſts in the *total extirpation of the articulation*, or the entire removal of the extremities of all the bones which form the joint, with the whole, or as much as poſſible, of the capſular ligament; thereby obtaining a cure by means of a callus, or by uniting the femur and tibia, when practiſed on the knee; and the humerus, radius, and ulna, when at the elbow, into one bone, without any moveable articulation.

The practicability of ſuch an operation, with a probability of ſucceſs, occurred to our author ſome years ago; but as the undertaking appeared liable to many objections he wiſhed to avoid being too precipitate in the attempt. The principal difficulties that occurred either from his own reflections, or the obſervations of his friends, were the hazard of wounding the principal blood veſſels;—the great inflammation, and large ſuppurations uſually conſequent on wounds of the articulations;—the uncertainty of of obtaining a firm callus;—the loſs of the infertions of the extenſor muſcles;—the doubt reſpecting the utility of the limb, provided a cure could be obtained;—the uncertainty of removing the whole diſeaſe where caries gave riſe to the operation;—and when undertaken on account of ſcrophulous affections of the joints, the hazard of a return of the ſame diſeaſe.

Theſe difficulties, though they might appear at firſt ſight very weighty, were found, on more attentive conſideration, to loſe much of their force. The danger of wounding the principal veſſels in the arm was very trifling, their ſituation being ſufficiently remote from the bone to place them out of all hazard. In the knee there was much more room for apprehenſion on this ſcore, the popliteal veſſels paſſing ſo immediately between the condyles of the femur. From an experiment, however, on the dead ſubject, it appeared that even theſe might be eaſily avoided. Mr. Park confeſſes that in this experiment, when the joint was removed, the appearance of the wound was ſomewhat formidable, exhibiting a very large cavity, with very thin parietes; and that there ſeemed little wanting to complete the amputation. Yet as the limb would not be deprived of any part of its nouriſhment, and every

 healhealing incifed furface, as well of bone as of foft parts, has a natural tendency to granulate, he could not fee any room to doubt that nature would find fufficient refources to repair this breach.

The next difficulty was the great inflammation, pain, and extenfive fuppurations, ufually confequent on wounds of large articulations; but as thefe appeared to be, in a great meafure, owing to the expofure of the capfular ligament, and of a large cartilaginous furface, and thefe parts were to be removed by the operation in queftion, the objection loft much of its weight.

The doubt of obtaining a firm callus was thought to have no reafonable foundation, as daily obfervation proves, that when two living furfaces of bare bone are oppofed to each other, they have ever a tendency to unite.

With refpect to the lofs of the infertions of the extenfor mufcles, it was thought fufficient to reply, that the joint being extirpated, there was no longer any want of mufcles to move it; and that the incifed ends of thefe mufcles as there would not be any part of them taken away, muft unavoidably attach themfelves to fome part of the callus; which was all that would be neceffary.

The

The queftion concerning the utility of the limb, provided a cure could be obtained, was, indeed, a very important one, and deferved well to be confidered. In the arm, however, the advantages arifing from the prefervation of a hand and fingers, with all their original motions, except thofe of pronation and fupination, were fo very evident, and fo very confiderable, as not to leave room for a moment's hefitation. In the leg, our author confeffes he was lefs fanguine in his expectations of advantages equal to the hazard, but ftill he was convinced that a ftiff limb would be infinitely preferable to an arti-ficial leg.

The two laft objections, viz. the uncertainty of removing the whole of the caries, and the danger of a return of the difeafe, feemed to operate with an equal degree of force againft amputation.

Upon the whole, Mr. Park could not fee any juft caufe to apprehend, that a perfon who had undergone an operation of this kind, would be in a lefs favourable ftate than one with a com-pound fracture with equal lofs of bone, but in which the principal blood veffels had efcaped unhurt. He at length, therefore, determined to put this operation in practice the firft favourable able opportunity, and it was not long before one occurred. A robuſt ſailor, aged 33, was admitted into the Liverpool Infirmary on account of a diſeaſed knee of ten years ſtanding. The joint was conſiderably enlarged, and the contraction of the flexor muſcles ſuch as to draw back the leg, ſo as to form a right angle with the thigh, in which poſition it was immoveably fixed. Every attempt to communicate to the joint the ſmalleſt degree of motion, gave him the moſt excruciating pain, and his health for ſome time had been rapidly declining. The nature of the propoſed operation was explained to him, and he very readily conſented to it in preference to amputation. In the performance of it one circumſtance occured, which our author has thought neceſſary to mention particularly, as it led him into ſome difficulty; which is, that he wiſhed to avoid making a tranſverſe inciſion, thinking it would be in his power, by a ſimple longitudinal one, after the patella was removed, to raiſe the integuments ſo as to divide the lateral and croſs ligaments, and after turning out the heads of the bones alternately, to ſaw off juſt as much as he might find diſeaſed. But in this he was greatly deceived. There appeared ſuch confuſion of parts on opening the the articulation, the ligaments being, in ſome parts, extremely thickened, in others in a ſloughy, ſuppurated ſtate, with the cartilages almoſt wholly deſtroyed, and the heads of the bones much eroded by the offenſive matter, of which there was a good deal in the joint; beſides, that ſome degree of bony union had already begun to take place between the head of the tibia and the inner condyle of the femur; that after ſpending ſome time in the attempt, it was thought adviſable to make a tranſverſe inciſion, and divide the femur above the condyles. An elaſtic ſpatula was introduced to guard the ſoft parts, while the bones were ſawed through. The quantity of bone removed was ſomewhat, though not much, more than two inches of the femur, and of the tibia rather more than one inch, which were but juſt enough to allow the leg to be brought into a right line with the thigh, the previous contraction of the flexor muſcles being ſuch as to keep the two ſawn ends of bone in cloſe contact. The only artery that was divided in the operation was one on the anterior part of the knee, which ceaſed to bleed before the operation was concluded; the ends of the bones, however, particularly that of the femur, bled pretty freely. As there remained a conſiderable redundance of integuintegument, it was ſupported by a few ſtitches. The lighteſt ſuperficial dreſſings only were applied, and the limb was placed in a caſe of tin ſufficiently long to receive the whole of it, from the ancle to the inſertion of the glutæus muſcle.

The patient paſt the day in a good deal of pain, had frequent vomitings, and loſt a good deal of blood, ſo that in the evening he was very languid, with a low weak pulſe of about 120. On looſening the bandages, which were full of blood, and become very tight and uneaſy, the hæmorrhage was found to have nearly ceaſed. The operation was performed on the firſt of July, and the diſcharge, which for ſome days was very conſiderable, as muſt have been expected from ſo large a ſurface of wound, was much diminiſhed by the 10th, and by the 21ſt was no more than ſufficient to moiſten the dreſſings. By this time likewiſe the cavity of the wound was in a great meaſure filled up, and the ends of the bones covered with granulations. Collections of matter were formed during the cure, but of theſe only two were of any importance, nor were theſe ſuch as to occaſion the ſmalleſt appearance of danger. It was pretty evident that they were occaſioned by a portion of diſeaſed capſular ligament, which was unavoidably avoidably left in the poſterior part. The confinement to bed was between nine and ten weeks, which was not a longer time than many compound fractures require. On the 31ſt of December the callus was ſtrong enough to allow the patient to raiſe the limb without the aſſiſtance of his hand, and at the time of writing the account he had got a ſtrong uſeful limb, free from pain or ſwelling, and was gone to ſea.

This is the only caſe in which our author has performed the operation in queſtion. It has convinced him, however, of its practicability and utility, and theſe, he thinks, in elbow caſes would be ſtill greater. But he wiſhes not to have it ſuppoſed that he is ſanguine enough to imagine, that the method he has recommended will certainly ſucceed in every caſe. He laments, that perſons labouring under diſeaſed joints are but ſeldom willing to ſubmit to any great operation till their lives are brought into imminent danger; in which ſtate amputation will be found the only reſource. To define, however, with accuracy the caſes in which extirpation will or will not be adviſable, he leaves to future experience to determine.

While this article was preparing for the preſs, we were informed, that ſince the publication of

Mr. Park's pamphlet, the operation he recommends has been successfully performed in an elbow case by a very ingenious and enterprizing surgeon of one of the Hospitals in London.

